# The effectiveness of continuous renal replacement therapy in critical COVID-19 patients with cytokine release syndrome: a retrospective, multicenter, descriptive study from Wuhan, China

**DOI:** 10.18632/aging.202838

**Published:** 2021-04-03

**Authors:** Huiling Xiang, Bin Song, Yuanyuan Zhang, Jianduan Zhang, Jing Xiong

**Affiliations:** 1 Department of Nephrology, Union Hospital, Tongji Medical College, Huazhong University of Science and Technology, Wuhan, China; 2 Department of Respiratory and Tuberculosis Disease, Wuhan Jinyintan Hospital, Wuhan, China; 3 Department of Maternal and Child Health, School of Public Health, Tongji Medical College, Huazhong University of Science and Technology, Wuhan, China

**Keywords:** COVID-19, cytokine release syndrome, kidney, C-reactive protein, continuous renal replacement therapy

## Abstract

Background: Coronavirus disease (COVID-19) has spread rapidly since 2019. Approximately 15% of the patients will develop severe complications such as multiple organ disease syndrome related to cytokine release syndrome (CRS). Continuous renal replacement therapy (CRRT) can remove inflammatory cytokines through filtration or adsorption. We evaluated the effectiveness of CRRT in COVID-19 patients with CRS.

Methods: This retrospective, multicenter, descriptive study included 83 patients with CRS from three hospitals in Wuhan.

Results: In COVID-19 patients with CRS, the fatality rate was even higher in CRRT group (P=0.005). However, inflammatory markers such as C-reactive protein, neutrophil counts, and D-dimer decreased after CRRT (P<0.05). Results of Lasso model showed that tracheotomy (β -1.31) and convalescent plasma (β -1.41) were the protective factors. In contrast, CRRT (β 1.07), respiratory failure (β 1.61), consolidation on lung CT (β 0.48), acute kidney injury (AKI) (β 0.47), and elevated neutrophil count (β 0.02) were the risk factors for death.

Conclusions: Our results showed that although CRRT significantly reduced the inflammation, it did not decrease the fatality rate of patients with CRS. Therefore, the choice of CRRT indication, dialysis time and dialysis mode should be more careful and accurate in COVID-19 patients with CRS.

## INTRODUCTION

An outbreak of coronavirus disease (COVID-19) in December 2019 in Wuhan, China, has developed into a global pandemic. COVID-19 is caused by infection with severe acute respiratory syndrome coronavirus-2 (SARS-CoV-2) virus [1]. Most patients with COVID-19 present with respiratory symptoms, including dyspnea and fever, and approximately 15% of patients develop severe complications including injury to the liver, heart, and kidneys [2, 3]. Although the treatment of COVID-19 has been closely investigated, no uniform clinical treatment protocol is available yet. Therefore, it is imperative that an effective treatment is found as soon as possible [4].

Cytokine release syndrome (CRS) plays a vital role in the occurrence and disease progression of the COVID-19 [5]. It has been confirmed that there is a substantial increase in inflammatory cytokines in COVID-19 patients in a critical condition, and this increase is related to the SARS-CoV-2 nucleic acids [6]. Acute respiratory distress syndrome (ARDS) is the main cause of death in COVID-19, associated with a massive inflammatory response mediated by the CRS [7]. SARS and Middle East respiratory syndrome (MERS) have also been shown to be associated with CRS and are closely linked to mortality [8]. CRS is a systemic inflammatory response that occurs mainly in cases of severe infection and is manifested by a sudden increase in the production of a large number of inflammatory factors, such as interleukin-2 (IL-2), IL-4, IL-6, and monocyte chemoattractant protein-1 [9]. Among these inflammatory cytokines, IL-1 and IL-6 are the main pathogenic factors. The cutoff value of specific inflammatory cytokines for CRS in patients with COVID-19 is yet to be defined. Recent research shows that an IL-6 value greater than 100 pg/mL carries the risk of death, and artificial-liver blood-purification therapy can be considered when IL-6 is five times the normal limit [6, 10].

Although there are no standard diagnostic criteria for CRS, some treatments based on managing CRS have been widely applied clinically. These include antagonism at the IL-6 receptor (using the antagonist Tocilizumab), artificial-liver blood-purification therapy that can remove cytokines from the circulation, and continuous renal replacement therapy (CRRT) can remove inflammatory factors such as cytokines from the circulation via filtration and adsorption [11]. Tocilizumab has been proved to be effective in COVID-19 at the initial stages [12, 13]. CRRT has been used in the treatment of COVID-19 patients with multiple organ failure, especially when accompanied by refractory CRS, and in some critical patients with acute kidney injury (AKI). One case report suggests that CRRT is initially effective in the treatment of patients with COVID-19 [14]. However, as little data on this topic exists, there is no definitive conclusion regarding the effectiveness of CRRT. In this study, we retrospectively collected and analyzed the clinical data of COVID-19 patients with CRS from three hospitals in Wuhan, to determine the effectiveness of CRRT.

## RESULTS

Of the 83 cases included in our study, 67 were classified as critical, and 16 as non-critical. Of the 67 critical patients, 38 cases were treated with CRRT during hospitalization. A total of 45 patients died, and 36 were cured or improved. The outcome of two patients is unknown.

### Patients demographics

The mean age of the patients was 67.3±12.6 years. Almost three-quarters (73.5%) of the patients were male; 67 were critical cases, and 13.3% of the patients had a history of smoking. The underlying medical conditions of the patients included hypertension (55.4%), diabetes (24.1%), tumors (6%), and chronic obstructive pulmonary disease (1.2%). Pulmonary CT revealed that 51.7% of the critical patients had consolidation, and 73.3 % of non-critical patients had ground-glass changes in the lungs. The mean peak creatinine value during hospitalization was 133.2 pg/mL (range, 72.8 to 276.7). AKI was present in 60.2%, MODS in 36.1%, and ARDS in 42.2% of the patients. Compared to the non-critical groups, the critical group had more patients with an IL-6 value >4000 pg/mL (29.9% vs.18.8%) (Table 1).

**Table 1:** The general characteristics of 83 COVID-19 patients by disease severity.

**Variables**		**Total(n=83)**	**Critical (n=67)**	**Non-critical (n=16)**	***t*/*χ^2^*/*Z***	***P***
Age (years)		67.3±12.6	67.6±12.3	65.9±13.9	-0.490^a^	0.625
Sex						
	Female	22(26.5)	16(23.9)	6(37.5)	0.630^c1^	0.427
	Male	61(73.5)	51(76.1)	10(62.5)		
Smoking		11(13.3)	8(15.7)	3(21.4)	0.011^c1^	0.916
Clinical outcomes						
	Cure/Improved	36(43.4)	22(33.3)	14(93.3)	17.820^c2^	<0.001
	Death	45(54.2)	44(66.7)	1(6.7)		
Underlying conditions						
	Diabetes	20 (24.1)	14(20.9)	6(37.5)	1.145^c1^	0.285
	Hypertension	46(55.4)	37(55.2)	9(56.2)	0.006^c2^	0.941
	Tumor	5(6.0)	5(7.5)	0(0.0)	-	0.578^f^
	COPD	1(1.2)	1(1.5)	0(0.0)	-	1.000^f^
Hospitalization time (days)		20(11,35)	19(11,30.75)^*^	34(22,47.5)^*^	683.0^b^	0.022
Laboratory test results						
	SO2 (%)	93(87,95)	92.5(85,95)^*^	93(88,95)^*^	413.5^b^	0.591
	PLT (10^9^/L)	158(107,216.5)	153(105.5,212.5)	180(135,233.25)	622.0^b^	0.324
	WBC count (10^9^/L)	8.21(6.32,11.84)	8.98(6.8,12.88)	5.58(3.68,7.08)	254.0^b^	0.001
	Neutrophil count (10^9^/L)	7.65(4.67,11.17)	8.01(5.48,12.01)	3.47(2.25,5.84)	217.0^b^	<0.001
	Neutrophil %	87.9(75,91.4)	89.9(80.1,92.1)^*^	68.85(63.42,77.65)^*^	94.5	<0.001
	Lymphocyte count (10^9^/L)	0.64(0.460.98)	0.59(0.42,0.96)	0.93(0.71,1.08)	741.5^b^	0.018
	Albumin (g/L)	30.6(26.2,33.95)	29.4(25.8,33.85)	33.75(30.68,36.1)	746.5^b^	0.015
	LDH (U/L)	375(263,534.5)	434(328.5,592)	248.5(217.5,266.25)	122.0^b^	<0.001
	Maximum creatinine value during hospitalization (μmol/L)	133.2(72.75,276.7)	146(92.45,291.35)	73.4(68.62,82.3)	312.0^b^	0.010
	high-sensitivity troponin I	26(9.5,111.75)	36(12.6,140.5)^*^	4.45(1.65,9.8)^*^	92.0^b^	<0.001
	D-dimer (ug/mL)	3.14(0.94,7.39)	3.31(0.88,8)^*^	2.67(1.66,4.97)	484.0^b^	0.672
	CRP (mg/L)	85.35(33.41,134.12)	96.7(43.8,143.8)^*^	14(2.58,49.5)^*^	202.0^b^	<0.001
	Ferritins (μg/L)	1271.82(726.64,2000)	1614(837,2000)^*^	565.1(303.82,957.31)^*^	80.0^b^	0.001
	PCT (ng/mL)	0.23(0.11,0.41)	0.24(0.12,0.41)^*^	0.06(0.05,0.26)^*^	206.0^b^	0.043
	Maximum IL-6 (pg/mL)					
	<P_25_ (<=250)	21(25.3)	15(22.4)	6(37.5)	-	0.426^c3^
	P_25_~P_75_ (250 -4000)	39(47.0)	32(47.8)	7(43.8)		
	>=P_75_(>=4000)	23(27.7)	20(29.9)	3(18.8)		
Lung CT main performance	Ground-glass	40(48.2)	29(48.3)	11(73.3)	3.013^c2^	0.083
	Consolidation	35(42.2)	31(51.7)	4(26.7)		
Treatments						
	CRRT	40(48.2)	38(56.7)	2(12.5)	10.144^c2^	0.001
	Antiviral	80(96.4)	64(98.5)	16(100.0)		1.000^c3^
	Tracheal cannula	56(67.5)	56(88.9)	0(0.0)	41.258^c1^	<0.001
	Tracheotomy	13(15.7)	13(21.3)	0(0.0)	2.275^c1^	0.131
	Antibiotic	73(88.0)	62(96.9)	11(68.8)	9.403^c1^	0.002
	Hormone	53(63.9)	47(78.3)	6(37.5)	8.139^c1^	0.004
	Globulin	41(49.4)	38(67.9)	3(18.8)	12.240^c1^	<0.001
	ACEI/ARB	6(7.2)	4(7.7)	2(14.3)		0.600^f^
	Convalescent plasma	10(12.0)	10(16.7)	0(0.0)	1.623^c1^	0.203
	Traditional Chinese medicine and pharmacy	27(32.5)	18(32.7)	9(69.2)	5.852^c2^	0.016
	High flow nasal catheter oxygen inhalation	63(75.9)	49(80.3)	14(87.5)	0.089^c1^	0.766
	Non-invasive ventilation	40(48.2)	40(65.6)	0(0.0)	21.834^c2^	<0.001
	Invasive mechanical ventilation	60(72.3)	59(89.4)	1(6.2)	41.212^c1^	<0.001
	ECMO	9(10.8)	9(14.3)	0(0.0)	1.358^c1^	0.244
Complications						
	AKI	50(60.2)	49(74.2)	1(6.2)	25.020^c2^	<0.001
	Respiratory failure	61(73.5)	57(91.9)	4(26.7)	27.417^c1^	<0.001
	Gastrointestinal bleeding	26(31.3)	25(45.5)	1(6.2)	8.208^c2^	0.004
	Acute liver dysfunction	45(54.2)	41(82.0)	4(25.0)	18.153^c2^	<0.001
	Acute myocardial injury	58(69.9)	56(88.9)	2(12.5)	34.337^c1^	<0.001
	ARDS	35(42.2)	35(62.5)	0(0.0)	19.459^c1^	<0.001
	MODS	30(36.1)	30(62.5)	0(0.0)	16.953^c1^	<0.001

Note: ^a^: t value by t test; ^b^: Z value by Wilcoxon rank sum test; ^c1^: continuity corrected *χ^2^* value by *χ^2^* test; ^c2^: *χ^2^* value by *χ^2^* test; ^f^: Fisher exact probability test p value.

*: contains missing values.

PLT: Blood platelets; WBC count: White blood cell count; SO2: Blood oxygen saturation; LDH: Lactate dehydrogenase; CRP: C-reactive protein; PCT: Procalcitonin; IL6: Interleukin 6; COPD: Chronic obstructive pulmonary disease; AKI: acute kidney injury.

Patients in the critical group had a significantly higher WBC count (P=0.001), neutrophil count (P<0.001), neutrophil percentage (P<0.001), LDH (P<0.001), peak creatinine (P=0.01), high-sensitivity troponin I (P<0.001), CRP (P<0.001), ferritin (P=0.001) and procalcitonin (PCT) (P=0.043) than those in the non-critical group. Patients in the critical group had significantly lower lymphocyte counts (P=0.018) and albumin levels (P=0.015) than patients in the non-critical group (Table 1).

Treatments such as CRRT (P=0.001), antibiotics (P=0.002), hormones (P=0.004), globulin therapy (P<0.001), invasive mechanical ventilation (P<0.001) and non-invasive ventilation (P<0.001), were each more commonly used in critical patients than in non-critical patients. Moreover, critical patients had a higher incidence of AKI (P<0.001), respiratory failure (P<0.001), ARDS (P<0.001), MODS (P<0.001), gastrointestinal bleeding (P=0.004), acute liver dysfunction (P<0.001) and acute myocardial injury (P<0.001) (Table 1).

### The role of CRRT in critical COVID-19 patients with CRS

Over half of the critical patients (38/67) were treated with CRRT. Those who received CRRT had a lower blood platelets (PLT) (P=0.023), and lower albumin (P=0.041), a higher peak creatinine (P=0.018), and a greater incidence of MODS (P<0.001) than those who did not receive CRRT. More patients receiving CRRT also underwent tracheal cannulation (P=0.003) or invasive mechanical ventilation (P=0.002). Most patients who received CRRT (64.7%) had pulmonary consolidations on CT (Table 2). Unexpectedly, mortality was higher in the CRRT group than that in the non-CRRT group (P=0.005).

**Table 2:** The general characteristics of 67 critical COVID-19 patients by CRRT.

**Variable**		**Total**	**CRRT (n=38)**	**Non-CRRT (n=29)**	***t*/*χ^2^*/*Z***	***P***
Age (years)		67.6±12.3	66.9±11.8	68.6±13.0	0.552^a^	0.583
Sex						
	Female	16	7(18.4)	9(31.0)	1.440^c2^	0.230
	Male	51	31(81.6)	20(69.0)		
Smoking		8	6(21.4)	2(8.7)		0.269^f^
Clinical outcomes						
	Cure/Improve	22	7(18.9)	15(51.7)	7.873^c2^	0.005
	Death	44	30(81.1)	14(48.3)		
Underlying conditions						
	Diabetes	14	7(18.4)	7(24.1)	0.325^c2^	0.568
	Hypertension	37	19(50.0)	18(62.1)	0.969^c2^	0.325
	Tumor	5	2(5.3)	3(10.3)		0.645^f^
	COPD	1	0(0.0)	1(3.4)		0.433^f^
Hospitalization time (days)		23.3±16.6	23.6±15.6^*^	23.0±18.1	477.5^b^	0.449
Laboratory test results						
	SO2 (%)	92.5(85,95)	90(84.5,95)^*^	93(88,95.5)^*^	462.5^b^	0.343
	PLT (10^9^/L)	153(105.5,212.5)	127(98.25,179)	185(112,247)	731.0^b^	0.023
	WBC count (10^9^/L)	9.9±4.4	9.8±3.9	10.0±4.9	0.180^a^	0.858
	Neutrophil count (10^9^/L)	8.80±4.30	8.80±3.89	8.80±4.87	-0.003^a^	0.997
	Neutrophil %	89.9(80.1,92.1)	89.95(86.6,93.13)^*^	89.8(78.5,91.4)	420.5^b^	0.321
	Lymphocyte count (10^9^/L)	0.59(0.42,0.96)	0.55(0.39,0.72)	0.64(0.48,1.12)	666.5^b^	0.146
	Albumin (g/L)	29.88±5.51	28.68±4.79	31.44±6.06	2.080^a^	0.041
	LDH (U/L)	434(328.5,592)	472(339.25,661)	430(321,502)	451.0^b^	0.208
	Maximum creatinine value during hospitalization (μmol/L)	146(92.45,291.35)	198.85(123.62,340.95)	132.7(63.1,214)	364.0^b^	0.018
	High-sensitivity troponin I	36(14.6,140.5)	46.6(19.9,184.25)^*^	23.7(12.55,70.82)^*^	354.0^b^	0.229
	D-dimer (ug/mL)	3.31(0.88,8)	2.26(0.92,8)^*^	3.53(0.88,6.98)	513.5^b^	0.915
	CRP (mg/L)	96.7(43.8,143.8)	97.76(54.34,143.57)^*^	89.16(33.93,143.8)	480.0^b^	0.583
	Ferritins (μg/L)	1614(837,20000)	1618.59(907.86,2000)^*^	1447.99(851.62,2000)^*^	183.0^b^	0.522
	PCT (ng/mL)	0.24(0.12,0.41)	0.3(0.14,0.49)^*^	0.23(0.12,0.27)^*^	276.5^b^	0.120
	Maximum IL-6 (pg/mL)					
	<P_25_ (<=250)	15	11(28.9)	4(13.8)	4.438^c2^	0.109
	P_25_~P_75_ (250 -4000)	32	14(36.8)	18(62.1)		
	>=P_75_(>=4000)	20	13(34.2)	7(24.1)		
Lung CT main performance	Ground glass	29	12(35.3)	17(65.4)	5.342^c2^	0.021
	Consolidation	31	22(64.7)	9(34.6)		
Treatments						
	Antiviral	64	35(97.2)	29(100.0)		1.000^f^
	Tracheal cannula	56	34(100.0)	22(75.9)		0.003^f^
	Tracheotomy	13	8(24.2)	5(17.9)	0.368^c2^	0.544
	Antibiotic	62	35(97.2)	27(96.4)		1.000^f^
	Hormone	47	30(83.3)	17(70.8)	1.326^c2^	0.250
	Globulin	38	24(77.4)	14(56.0)	2.911^c2^	0.088
	ACEI/ARB	4	1(3.3)	3(13.6)		0.299^f^
	Convalescent plasma	10	7(21.2)	3(11.1)	0.485^c1^	0.486
	Traditional Chinese medicine and pharmacy	18	8(25.8)	10(41.7)	1.546^c2^	0.214
	High flow nasal catheter oxygen inhalation	49	25(75.8)	24(85.7)	0.950^c2^	0.330
	Non-invasive ventilation	40	23(65.7)	17(65.4)	0.001^c2^	0.979
	Invasive mechanical ventilation	59	37(100.0)	22(75.9)		0.002^f^
	ECMO	9	6(17.1)	3(10.7)	0.131^c1^	0.717
Complications						
	AKI	49	30(81.1)	19(65.5)	2.059^c2^	0.151
	Respiratory failure	57	35(94.6)	22(88.0)		0.385^f^
	Gastrointestinal bleeding	25	16(48.5)	9(40.9)	0.306^c2^	0.580
	Acute liver dysfunction	41	26(81.2)	15(83.3)	0.000^c1^	1.000
	Acute myocardial injury	56	32(88.9)	24(88.9)		1.000^f^
	ARDS	35	25(71.4)	10(47.6)	3.175^c2^	0.075
	MODS	30	26(81.2)	4(25.0)	14.400^c2^	<0.001

Note: ^a^: t value by t test; ^b^: Z value by Wilcoxon rank sum test; ^c1^: continuity corrected *χ^2^* value by *χ^2^* test; ^c2^: *χ^2^* value by *χ^2^* test; ^f^: Fisher exact probability test p value.

*: contains missing values.

PLT: Blood platelets; WBC count: White blood cell count; SO2: Blood oxygen saturation; LDH: Lactate dehydrogenase; CRP: C-reactive protein; PCT: Procalcitonin; IL6: Interleukin 6; COPD: Chronic obstructive pulmonary disease; AKI: acute kidney injury.

Compared to the non-CRRT group, the CRRT group had more patients with an IL-6 value >4000 pg/mL (24.1% vs. 34.2%). The blood oxygen saturation in patients who received CRRT was lower than in the non-CRRT group [90 (84.5,95)% vs.93 (88,95.5)%] and the lymphocyte count [0.55 (0.39,0.72) 10^9^g/L vs. 0.64 (0.48,1.12) 10^9^g/L] followed the same pattern, demonstrating lower values in the CRRT patients. The CRP was higher in the CRRT than in the non-CRRT group [97.7 (54.34,143.57) mg/L vs. 89.16 (33.93,143.8) mg/L)]. Moreover, the incidence of AKI and ARDS in critical patients treated with CRRT was 81.1% and 71.4%, respectively, which was higher than the corresponding figures of non-CRRT (Table 2). Compared with the non-CRRT patients, the patients in the CRRT group appeared to be in a worse condition, although there was no statistical difference in the indicators mentioned above.

For the 38 patients treated with CRRT, the changes of inflammation-related indicators before and after CRRT were compared. These included the count and percentage of neutrophils, WBC counts, lymphocytes counts, and levels of IL-6, CRP, D-dimer, and PCT. The analysis showed that after CRRT, WBC counts (P=0.039), neutrophil counts (P=0.014), CRP (P=0.049), D-dimer (P=0.006) all declined significantly from the values before CRRT. However, lymphocytes, PCT and IL-6 did not change significantly (Table 3).

**Table 3:** The effect of CRRT on inflammatory response.

**Variable**	**Before**	**After**	**Difference**	**V**	***P***	**N**
WBC count(10^9^/L)	12.85(9.96,18.77)	8.42(4.44,15.06)	-4.43(-10.12,2.14)	176.5	0.039	34
Neutrophil counts	12.22(8.45,17.71)	7(3.47,12.19)	-4.24(-9.65,1.47)	134	0.014	32
Neutrophil %	92.2(88.88,94.80)	89(79.95,92.52)	-1.95(-5.05,0.62)	177	0.040	34
Lymphocyte counts (10^9^/L)	0.54(0.36,0.96)	0.54(0.24,1.02)	-0.1(-0.28,0.20)	249	0.412	34
CRP (mg/L)	120.10(64.66,160)	63.6(42.11,128)	-37.1(-68.34,33.58)	126	0.049	31
PCT (ng/mL)	1.67(0.70,4.69)	2.58(0.69,6.50)	-0.86(-2.39,4.74)	246	0.747	32
D-dimer (ug/L)	7.08(2.29,12.14)	3.94(1.88,7.18)	-2.61(-6.04,0)	90	0.006	31
IL 6 (pg/L)	20.95(9.13,91.29)	30.34(16.08,416.02)	9.30(-13.75,287.23)	175	0.267	24

Note: V is the statistic of Wilcoxon signed rank test. N is the number of the patients.

WBC count: White blood cell count; CRP: C-reactive protein; PCT: Procalcitonin; IL6: Interleukin 6.

### Factors related to mortality

The results from Lasso analysis indicated that patients with CRRT (β 1.07), consolidation of the lungs (β 0.48), respiratory failure (β 1.61), AKI (β 0.47), and elevated neutrophils (β 0.02) could have a higher the risk of death. Tracheotomy (β -1.31) and convalescent plasma (β -1.41) were found to be negatively correlated with death (Table 4).

**Table 4:** The results of lasso regression model on the death outcome of the 66 critical patients with COVID-19.

**Variables**	**β**	OR^*^
CRRT	1.07	2.92
Tracheotomy	-1.31	0.27
Convalescent plasma	-1.41	0.24
Respiratory failure	1.61	4.99
Lung CT main performance	0.48	1.62
AKI	0.47	1.60
Neutrophil %	0.02	1.02

Note: AKI: acute kidney injury; WBC count: White blood cell count; LDH: Lactate dehydrogenase; CRP: C-reactive protein; *: OR value was calculated based on β, OR=e^β^. Variables with positive β are risk factors of death outcome while those negative are protective.

In addition to the variables shown in the Table 4, these variables such as cough, dyspnea, hormone treatment, Invasive mechanical ventilation, acute myocardial injury, WBC count (10^9^/L), Neutrophil count (10^9^/L), Lymphocyte count (10^9^/L), Albumin (g/L), LDH (U/L), maximum creatinine value during hospitalization(μmol/L), CRP (mg/L) and D-dimer (ug/mL) were also included into lasso model. However, their coefficients were shank to 0 by the model due to their negligible effects, therefore these variables were not included in the table.

Logistic regression analysis of 66 critical patients further showed that respiratory failure was an independent risk factor for death (odds ratio [OR], 0.06; 95% confidence interval [CI], 0.01-0.38, P<0.001), while tracheotomy was found to be an independent protective factor for death (OR, 68.72; 95% CI, 4.81-10404.57, P<0.001). Therefore, it is suggested that timely tracheotomy should be performed in critically ill patients with respiratory failure (Table 5).

**Table 5:** Results of the exact logistic regression on the death outcome of the 66 critical patients with COVID-19.

	**coef**	**se(coef)**	**lower 0.95**	**upper 0.95**	***χ^2^***	***P***	**OR (95%CI)**
Tracheotomy(Yes)	- 2.73	0.99	- 4.80	- 0.97	9.45	<0.001	0.06 (0.01,0.38)
Respiratory failure(Yes)	4.23	1.87	1.57	9.25	10.79	<0.001	68.72 (4.81,10404.57)

## DISCUSSION

In this study, we found that the fatality rate of critical COVID-19 patients with CRS who received CRRT was higher than that of those who did not. This means that the clearance of inflammatory factors by CRRT did not improve the prognosis of CRS. Furthermore, the results from Lasso analysis also showed that CRRT was a dependent risk factor for death. The result may not have been expected, and is not consistent with some reports in the literature. For example, several case reports suggest that CRRT can improve the prognosis and may be a potential treatment for COVID-19 [15–17]. However, the results of Fominskiy E et al. also show that CRRT does not reduce the mortality of patients with COVID-19, and CRRT also carries a higher risk of in-hospital death [18]. Therefore, whether CRRT can improve the prognosis of critical COVID-19 patients with CRS is still a controversial question.

In our study, the patients in the CRRT group were more seriously ill. Compared with the non-CRRT patients, they had a higher level of peak creatinine and had a higher incidence of MODS or ARDS. In addition, the unsuitable choice of dialysis timings or dialysis mode, and dialysis- related complications, such as bleeding caused by anticoagulation, might contribute to the high fatality rate in the CRRT group [19]. The therapeutic effect of CRRT is reduced when IL-6 suddenly increases to more than 100 pg/mL or when other complications are present [6, 20]. Therefore, when COVID-19 patients develop CRS, the use of CRRT should not be delayed. As currently recommended for interventional treatment of artificial-liver blood-purification, when IL-6 is five times the normal limit, the application of CRRT should be considered. If the characteristics of our cases and the current literature are considered together, IL-6 values greater than 100pg/ml could be an important critical index for intervention. However, the determination of the specific cutoff value of IL-6 for early intervention with CRRT requires further investigation [10]. Although CRRT did not improve the survival of critical COVID-19 patients with CRS, some inflammatory markers such as the CRP, D-dimer, WBC counts, and neutrophil counts decreased significantly after CRRT. It is suggested that CRRT can reduce inflammation in such patients.

The results from Lasso analysis indicated that AKI is a risk factor for death in critical COVID-19 patients with CRS. This result is consistent with the conclusion of a study conducted by Tongji Hospital, another hospital focused on the treatment of COVID-19 patients in Wuhan [21]. The incidence of AKI in our study was much higher than previously reported for ICU patients [2]. It is suggested that in addition to the lung, the kidney is also one of the most frequently affected organs in COVID-19 infections. Recent studies have confirmed that the expression of angiotensin-converting enzyme 2, a cell entry receptor of SARS-CoV-2, is very high in the kidney [22, 23]. In addition, the changes in hemodynamics and the damage done by inflammatory cytokines to the kidneys are also the reason for the high incidence of AKI in COVID-19 patients [24, 25]. So, the urinary system is a potential route for SARS-CoV-2 infection. In addition, the Lasso analysis suggests that consolidation on lung CT and elevated neutrophils also increase the risk of death.

The incidence of respiratory failure is higher in patients with critical COVID-19 (73.5%), and multiple regression analysis also confirms that respiratory failure is a risk factor for death. Tracheotomy was found to be protective for death among critical COVID-19 patients with CRS, indicating that patients with respiratory failure should undergo tracheotomy and invasive ventilation at the correct time.

In terms of treatment, in addition to CRRT and invasive ventilation, many other methods are used to treat these seriously ill patients. These treatments include convalescent plasma, hormones, globulin, and Chinese medicine, but the benefits are also controversial. In our study, convalescent plasma treatment might have been beneficial for critical COVID-19 patients with CRS, according to Lasso analysis. Hormones and globulin were commonly used treatments in severe SARS or MERS patients in the past. Over 78.3% of the critical patients in this study received hormone therapy for the treatment of COVID-19. Though the treatment with hormones was not found to be either a protective or a risk factor in this study, it should be carefully considered [26]. Regarding Chinese medicine, we did not see any protective factors in our results, which may be inconsistent with some reports [27]. The reason may be that most Chinese medicines are used in patients with mild or moderate symptoms, while most COVID-19 patients with CRS have severe or critical symptoms, and fewer of them are treated with Chinese medicine.

Our study has several limitations. First, though we have compiled cases from three hospitals that mainly treated patients with COVID-19, the sample size is still comparatively small. Second, although some confounding factors were compensated for, the influences of some unknown/unavailable factors cannot be completely excluded. Third, the medical records of two patients are incomplete, and the prognosis is unknown, resulting in missing data.

In conclusion, the fatality rate of CRRT patients did not decrease as expected, and even had an opposite trend in our study. The decision whether and how to use CRRT in COVID-19 patients with CRS should be carefully assessed. In the future, more studies and larger sample size are needed to evaluate the effect of CRRT on COVID-19 patients with CRS. Convalescent plasma therapy might be clinically considered in critical COVID-19 patients, and tracheotomy could be recommended for those who developed respiratory failure.

## MATERIALS AND METHODS

### Study design

In this retrospective, multi-center study, we analyzed results from 83 patients diagnosed with COVID-19 and CRS from December 2019 to July 2020, at three participating hospitals (Wuhan Union hospital, Wuhan Jinyintan Hospital, and Wuhan First Hospital). The following inclusion criteria were applied: a laboratory diagnosis of COVID-19 (a positive throat swab nucleic acid test or positive serum COVID-19 specific antibody test), and a peak IL-6 value >100 pg/mL, or a peak IL-6 value of 50-100 pg/mL with concurrent ARDS or multiple organ disease syndrome (MODS). Clinical indications for CRRT include hyperkalemia, acidosis, multiple organ dysfunction, or severe CRS. This study was approved by the institutional ethics board of Wuhan First Hospital (W202003–2) and conducted in accordance with the Declaration of Helsinki. The requirement for informed consent was waived by the ethics board.

### Definition

According to the New Coronavirus Pneumonia Prevention and Control Plan (seventh edition) published by the National Health Commission of China, COVID-19 can be classified as mild, moderate, severe, and critical. Mild infections are those that only have clinical manifestations but no abnormalities on computed tomography (CT) scan of the lungs. Patients with clinical and pulmonary CT manifestations are considered as moderate cases. Severe cases of COVID-19 are defined as those with a respiratory rate ≥ 30 breaths/min, blood oxygen saturation ≤ 93%, or arterial PO_2_/oxygen concentration ≤ 300 mmHg. A patient with COVID-19 is considered to be critical when respiratory failure requires mechanical ventilation, or the patient experiences shock or multiple organ failure and is transferred to the intensive care unit. After careful evaluation, all the patients included in this study were classified as severe and critical cases and therefore were divided into critical and non-critical groups.

AKI was defined as an increase in serum creatinine by 26.52 mmol/L within 48 hours or by more than 50% from the baseline within 7 days [28].

### Data collection

After careful review, data, including demographics, clinical characteristics, laboratory and radiological examinations and treatments, were extracted from the patients’ medical records. Patients were classified as critical or non-critical, according to the criteria described in the definitions sections above. We retrieved the values of relevant CRRT indicators obtained before and after CRRT. All laboratory examinations of patients were carried out by trained physicians.

### Statistical analyses

Categorical variables were presented as numbers and proportions, and the difference between the groups was determined using the chi-square test or Fisher's exact test. Continuous variables were presented as mean (SD) or median [interquartile (IQR)], and differences between the groups were determined using a two-sample t-test or Wilcoxon rank-sum test. Lasso and accurate logistic analysis were conducted to identify the factors related to death. The explanatory variables included treatments (CRRT, tracheotomy, convalescent plasma), respiratory failure, consolidation on lung CT, AKI, elevated neutrophil percentage, cough, dyspnea, hormone treatment, invasive mechanical ventilation, acute myocardial injury, White blood cell (WBC) count (x 10^9^/L), neutrophil count (x 10^9^/L), lymphocyte count (x 10^9^/L), albumin (g/L), lactate dehydrogenase (LDH) (U/L), maximum creatinine value during hospitalization (μmol/L), C-reactive protein (CRP) (mg/L) and D-dimer (ug/mL) levels. Statistical significance was set as two-sided with P < 0.05. All the analyses were conducted using R software (version 3.6.2, R Foundation), and the Lasso and acute logistic analysis were performed with the glmnet and logistf package.

### Data availability statement

The data underlying this article are available in the article and its online supplementary material.
